# Electrocatalytic Determination of Uric Acid with the Poly(Tartrazine)-Modified Pencil Graphite Electrode in Human Serum and Artificial Urine

**DOI:** 10.1021/acsomega.3c02561

**Published:** 2023-09-11

**Authors:** Lokman Liv, Merve Portakal, Meryem Sıla Çukur, Beyza Topaçlı, Berkay Uzun

**Affiliations:** † Electrochemistry Laboratory, Chemistry Group, The Scientific and Technological Research Council of Turkey, National Metrology Institute, (TUBITAK UME), 41470 Gebze, Kocaeli, Turkey; ‡ Faculty of Technology, Department of Biomedical Engineering, Pamukkale University, 20160 Denizli, Turkey; § Faculty of Technology, Department of Biomedical Engineering, Kocaeli University, İzmit, 41380 Kocaeli, Turkey; ∥ School of Engineering, Department of Biomedical Engineering, TOBB University of Economics and Technology, 06560 Ankara, Turkey

## Abstract

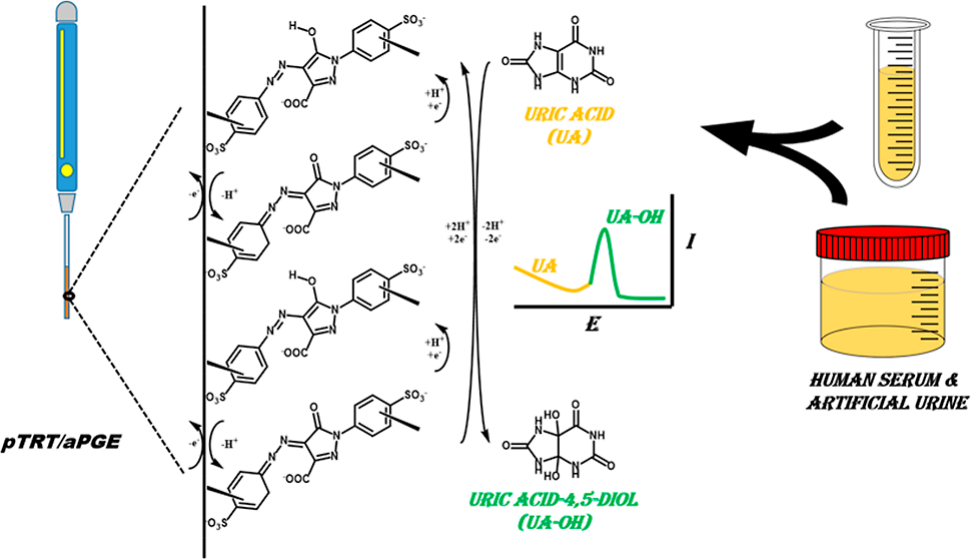

A novel electrocatalytic sensing strategy was built for uric acid (UA) determination with an exceptionally developed poly(tartrazine)-modified activated pencil graphite electrode (pTRT/aPGE) in human serum and artificial urine. The oxidation signal of UA at 275 mV in pH 7.5 phosphate buffer solution served as the analytical response. Cyclic voltammetry, electrochemical impedance spectroscopy, scanning electron microscopy, energy-dispersive X-ray spectroscopy, and X-ray photoelectron spectroscopy were used to characterize the sensing platform, which was able to detect 0.10 μM of UA in the ranges of 0.34–60 and 70–140 μM. The samples of human serum and artificial urine were analyzed by both the pTRT/aPGE and the uricase-modified screen-printed electrode. The results were statistically evaluated and compared with each other within the confidence level of 95%, and no significant difference between the results was found.

## 1 Introduction

Uric acid (2,6,8-trihydroxypurine, UA) is the end product of purine metabolism in humans, formed by the oxidation of xanthine and hypoxanthine, but is more toxic than these^1^ and is omnipresent in urine and blood.^2^ The normal range of UA in healthy individuals varies from 1.4 to 4.4 mM in urine and from 240 to 520 μM in blood.^3^ UA is a natural antioxidant and is able to eliminate free radicals and singlet oxygen. It also binds iron, preventing iron-dependent ascorbate oxidation. Due to these properties, UA hinders the destruction of human tissues and cells.^4^ High level of UA may lead to Lesch-Nyhan syndrome, hyperuricemia, obesity, gout, diabetes, kidney failure, and cardiovascular disease, while low level of UA could be linked to Alzheimer, polyarthritis, Parkinson, acromegaly, multiple sclerosis, and yellow fever atrophy.^3−5^ In addition, a low level of UA might be related to a deficiency of molybdenum and toxicity of copper, and an anomalous low UA level may indicate Wilson’s disease and Fanconi’s disease.^6^ Therefore, monitoring the level of UA in human urine or blood is of great importance and pivotal indicator in various fields involving clinical diagnostics, health assessment and monitoring, and biological analysis.

Numerous methods of determining UA have been reported, including ones based on capillary electrophoresis,^7^ chemiluminescence,^8,9^ UV–visible spectrophotometry,^10,11^ fluorescence spectroscopy,^12,13^ chromatography,^14−16^ and electroanalytical methods.^1−6,17−27^ The method based on capillary electrophoresis has a high limit of detection (LOD, i.e., 333 nM) and a long analysis time (i.e., 14 min).^7^ In chemiluminescence-based methods,^8,9^ there are disadvantages including complex procedures, time-consuming steps for system preparation, and analysis time, and the usage of uricase enzyme^8^ and porcine liver.^9^ In UV–visible spectrophotometric methods, both of them have complex procedures and LODs at the high magnitudes of nM (i.e., 476 nM)^10^ and μM (i.e., 3.14 μM),^11^ one of them requires a column for preconcentration and purification at sample preparation step,^10^ and the other needs a long time (i.e., 2 h), high temperature (i.e., 160 °C), and enzyme (i.e., uricase) for probe synthesis.^11^ The methods based on fluorescence spectroscopy have long probe preparation (i.e., >2 d,^12^ and >2 h^13^) and analysis time (i.e., 45 min^12^ and 25 min^13^) and require some enzymes such as uricase and horseradish peroxidase.^12,13^ When the methods based on the chromatography technique are examined, using expensive instruments and large amounts of chemicals^14−16,28^ are among the common disadvantages. On the other hand, there are also method-specific disadvantages such as high LOD value (i.e., 125 nM,^14^ 238 nM,^15^ and 19.63 μM^16^), presence of high-temperature process (i.e., 350 °C),^14^ and long analysis time (i.e., 15 min).^15,16^ However, owing to several noticeable features of electrochemical methods involving ease of fabrication and miniaturization, low cost, portability, quick response, notable sensitivity and selectivity, they have been identified to be more potent than other techniques for determining UA.

A wide variety of chemically modified electrodes including methylcellulose/graphene oxide/iron oxide nano hydrogel/glassy carbon electrode,^1^ β-cyclodextrin/reduced graphene oxide/screen printed electrode,^2^ (platinum nanoparticles)-graphene flakes-flavin mononucleotide/gold interdigitated microelectrode,^3^ multiwalled carbon nanotubes/poly(4-amino-3-hydroxy naphthalene sulfonic acid)/glassy carbon electrode,^4^ acrylic acid-ethylene glycol dimethacrylate-2,2′-azobis(2-isobutyro) nitrile/carbon paste electrode,^5^ nickel ferrite/glassy carbon electrode,^6^ platinum nanosheets/fullerene/glassy carbon electrode,^21^ nickel ferrite nanorods/sulfur-doped carbon nanoparticles/glassy carbon electrode,^22^ poly(solid red a)/carbon nanotube paste electrode,^23^ N,P-doped hollow mesoporous carbon nanospheres-phytic acid/glassy carbon electrode,^24^ SnO_2_/graphene/glassy carbon electrode,^25^ three-dimensional porous graphene/glassy carbon electrode,^26^ poly(glyoxal-bis(2-hydroxyanil))/glassy carbon electrode,^27^ nickel hydroxide/solar graphene/glassy carbon electrode,^17^ platinum nanoparticles/multiwalled carbon nanotubes/glassy carbon electrode,^18^ copper nanoparticles/polypyrrole/glassy carbon electrode,^19^ poly(*p*-aminophenol)/glassy carbon electrode,^20^ gold clusters/*N*-acetyl-l-cysteine–multiwalled carbon nanotubes/glassy carbon electrode,^29^ gold-silver bimetallic nanoparticles/graphene oxide/thionine/glassy carbon electrode,^30^ urate oxidase/metal organic frameworks (gold nanocages)/glassy carbon electrode,^31^ and urate oxidase-cobalt-based metal–organic framework/boron nanosheets-doxorubicin/glassy carbon electrode^32^ have been reported for determining UA and are summarized in Table S1.

Chemically modified electrodes are known to improve electron-transfer kinetics over bare electrodes.^33,34^ Among them, polymer film-modified electrodes provide advantages such as good adhesion to the surface, improved peak current, large effective surface area, worthy ionic and electronic conductivity, catalytic effect, and good selectivity and sensitivity.^4,27,35−100^ Due to these properties, polymer film electrodes are widely used in the development and application of electrochemical sensors and for the determination of many biologically active species.^27,37,38^

Our research motivation is to create an electrochemical sensing platform that addresses the limitations found in the literature, such as the reliance on costly supporting materials like glassy carbon electrodes, the time-consuming and multi-step sensor preparation processes, and the limited availability of disposable platforms. To achieve this goal, a cost-effective and disposable pencil lead was employed, enabling the development of a polymer film-modified electrode in a single step for the determination of UA. In parallel with addressing these issues, our aim is also to provide sensitivity, accuracy, and selectivity that are on par with the methods described in the literature.

To the best of our knowledge, this is the first example of electrochemical polymerization of tartrazine onto the pencil graphite electrode (PGE). The poly(tartrazine) film was characterized by cyclic voltammetry (CV), electrochemical impedance spectroscopy (EIS), scanning electron microscopy (SEM), energy-dispersive X-ray spectroscopy (EDX), and X-ray photoelectron spectroscopy (XPS) techniques, and the produced platform was successfully used for determining UA in human serum and artificial urine samples.

## 2 Materials and Methods

### 2.1 Chemicals and Equipment

Tartrazine (Alfa Aesar A17682, 98%), UA (Alfa Aesar A13346, 99%), acetic acid (J.T. Baker 6003, 99–100%), sodium hydroxide (Merck 1.06498, Emsure, ≥99%), potassium dihydrogen phosphate (Merck 1.04873, Emsure, ≥99.5%), potassium chloride (Sigma-Aldrich P3911, ACS Reagent, 99.0–100.5%), sucrose (Sigma-Aldrich S0389, ≥99.5%), d-glucose (Sigma-Aldrich G8270, ≥99.5%), dopamine hydrochloride (TCI A0305, >98.0%), glycine (Merck 1.04201, ≥99.7%), citric acid (Merck 8.18707, ≥99.0%), sodium chloride (Merck 1.06406, Suprapur, 99.99%), sodium nitrite (Merck 1.06549, Emsure, ≥99.0%), sodium perchlorate (Sigma-Aldrich 410241, ACS Reagent, ≥98.0%), calcium chloride (Alfa Aesar L13191, 97%), l-cysteine (Sigma-Aldrich C7352, ≥98%), iron(III) chloride (Sigma-Aldrich 8.03945), aluminum chloride (Alfa Aesar A11892, 99%), magnesium chloride (Sigma-Aldrich M8266, ≥98.0%), l-ascorbic acid (Sigma-Aldrich A5960, BioXtra, ≥99.0%), sodium sulfate (Merck 1.06643, Emprove, 99.0–100.5%), and sodium nitrate (Sigma-Aldrich S5506, ReagentPlus, ≥99.0%) were used as analytical reagent grade. All solutions were prepared in ultrapure water and stored in high-density polyethylene falcon tubes, and the solutions of tartrazine and UA were prepared daily.

Voltammetric measurements were carried out with an EIS module combined DropSens potentiostat/galvanostat using a standard three-electrode system consisting of a poly(tartrazine)-modified activated pencil graphite electrode (pTRT/aPGE, pencil body: Rotring Tikky, supporting surface: Tombow 2B 0.7 mm, surface area: 22.36 mm^2^, immersion depth: 1 cm), an Ag/AgCl electrode (inner solution: 3 M NaCl, BASi MF-2052 RE-5B), and a platinum wire (BASi MW-1032, 7.5 cm) as the working, reference, and auxiliary electrodes, respectively. All graphite grades, H, HB, B, and 2B, were purchased from a stationary in Kocaeli.

An uricase-modified screen-printed electrode (DropSens Ref. UA10) was used for comparison measurements in human serum and artificial urine samples. A Mettler Toledo Seven Compact pH meter with InLab Routine Pro-ISM probe was used to prepare buffer solutions. Ultrapure water was obtained by the Merck Millipore Milli-Q Direct 8 system. SEM analyses were performed with a Hitachi Schottky SU5000 field emission-scanning electron microscope with 15 kV of voltage, 30 of spot intensity (relative amount), and a SE(L) detector. EDX analyses were carried out with a FEI Oxford Instruments model 7260 EDX with 15 kV of voltage and 10^4^ μm^2^ of area, and AZtec software was operated by using mass percentages. XPS measurements were applied with a Thermo Fisher K-Alpha XPS with 300 μm of spot size, 50 eV of pass energy, 0.1 eV of energy step size, and an Al Kα gun. The XPS instrument is equipped with clean internal standard samples (copper, silver, and gold) that serve the purpose of automatically calibrating the XPS-binding energy (BE) scale. The calibration process involved using reference lines from Au 4f_7/2_ (84.1 eV), Cu 2p_3/2_ (932.2 eV), and Ag 3d_5/2_ (368.2 eV) to ensure accurate measurement of BEs.

### 2.2 Preparation of pTRT/aPGE

**Figure 1:**
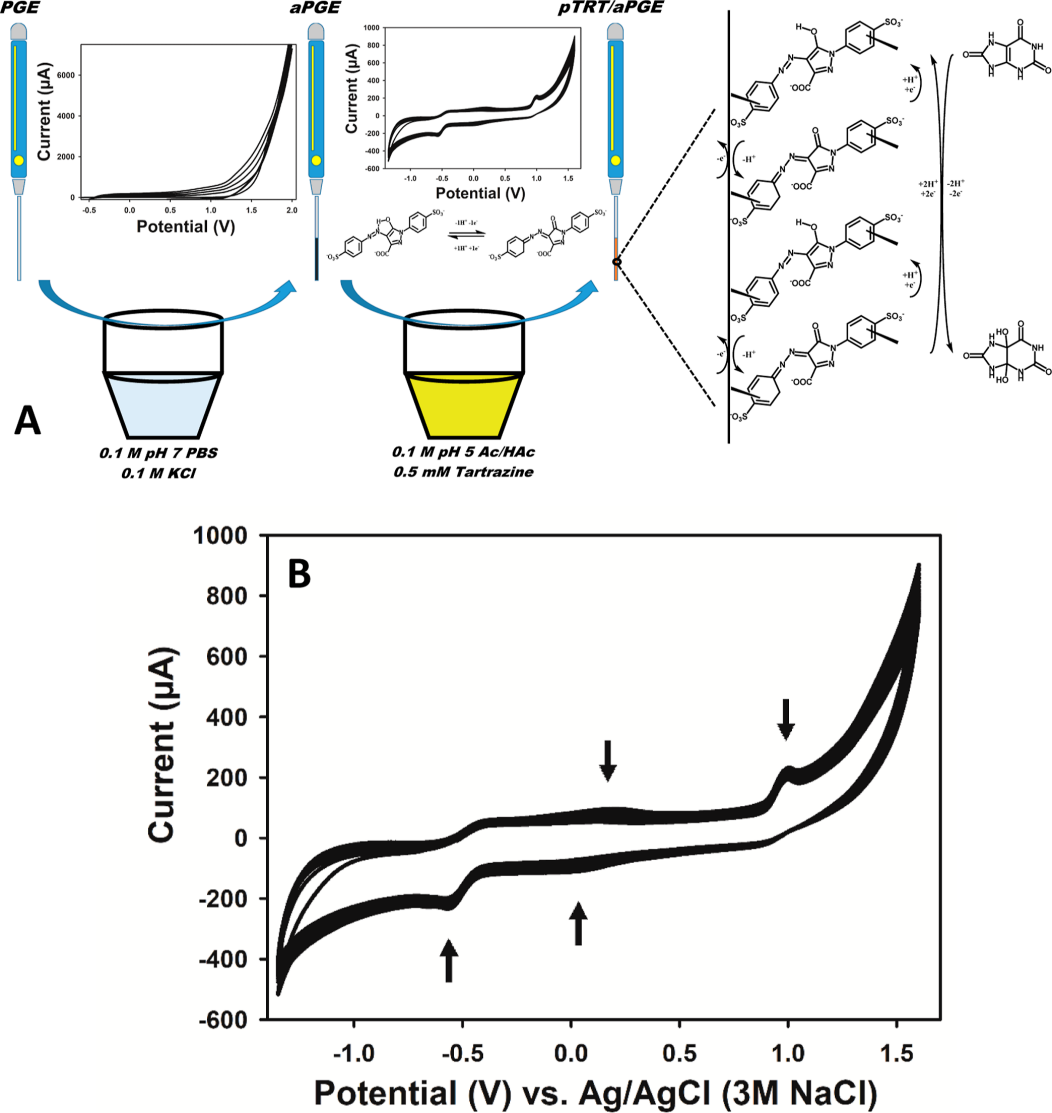
(A) Preparation procedure of pTRT/aPGE and possible mechanism of electrochemical detection and (B) electro-polymerization voltammograms of pTRT/aPGE.

The process of preparing pTRT/aPGE, electro-polymerization voltammograms of the polymer film-modified electrode, and possible electrochemical detection mechanism of UA are illustrated in Figure 1A,B, respectively. The preparation of pTRT/aPGE consists of two steps including activation of bare PGE and electro-polymerization of tartrazine onto activated PGE, respectively. For this purpose, the pTRT film was coated in 0.5 mM of tartrazine and 0.1 M of pH 5 acetic acid-acetate buffer solution via cycling 15 repetitive scans between −1.35 and 1.60 V with a scan rate of 100 mV/s after PGE was activated^39^ in 0.1 M of pH 7 phosphate buffer solution (PBS) and 0.1 M KCl via cycling five times between −0.6 and 2.0 V with 50 mV/s of scan rate, and the final volume of the solution was set to 10 mL in both cases. The increase and decrease of the peak current values in the respective regions proportional to the number of cycles indicate that the electro-polymerization has occurred, as shown in Figure 1B. The obtained platform was denoted as pTRT/aPGE and prepared daily.

### 2.3 Voltammetric Measurement Procedure

The potential was scanned from −0.4 to 1.0 V using the differential pulse voltammetry (DPV) mode with 5 mV of step amplitude, 25 mV of pulse amplitude, and a scan rate of 25 mV/s. CV measurements were applied in the range of −1 to 1 V (−0.7 to 1.1 V for characterization in the solution of 1 mM of K_3_[Fe(CN)_6_], 1 mM of K_4_[Fe(CN)_6_], and 0.1 M of KCl) with a step amplitude of 3 mV and a proper scan rate. The solution consisting of 0.05 M pH 7.5 PBS with or without real samples (i.e., human serum and artificial urine) was set to 10 mL with ultrapure water and deaerated with argon for 3 min.

### 2.4 Amperometric Measurement Procedure

Amperometric determination of UA in human serum and artificial urine samples was performed in 0.02 M pH 8 borate buffer solution with a set potential of −100 mV, an interval time of 0.5 s, and a commercial uricase-modified screen-printed electrode.

### 2.5 Sample Preparation Procedure

Human serum samples were obtained from healthy volunteer individuals and those were tested for anti-HCV, anti-HIV-1/2, HBsAg, HBV DNA, HCV RNA, HIV RNA, and syphilis and were found to be negative. An artificial urine sample was prepared in accordance with the literature.^40^ An external calibration method for artificial urine (200 μM of UA was added to the bulk sample; 50-fold diluted sample, 0.2 mL sample/10 mL for the proposed method; 4-fold diluted sample, 12.5 μL sample/50 μL for the amperometric method) and human serum (50-fold diluted sample, 0.2 mL sample/10 mL for the proposed method; 4-fold diluted sample, 12.5 μL sample/50 μL for the amperometric method) was used to determine either the added or the content of UA with the proposed and the amperometric method.

In addition, recovery experiments were performed for human serum (50-fold diluted sample) and artificial human urine (10-fold diluted sample) samples spiked with 10, 25, 50, 80, 100, and 130 μM of UA.

## 3 Results and Discussion

### 3.1 Surface Characterization of the pTRT/aPGE

**Figure 2:**
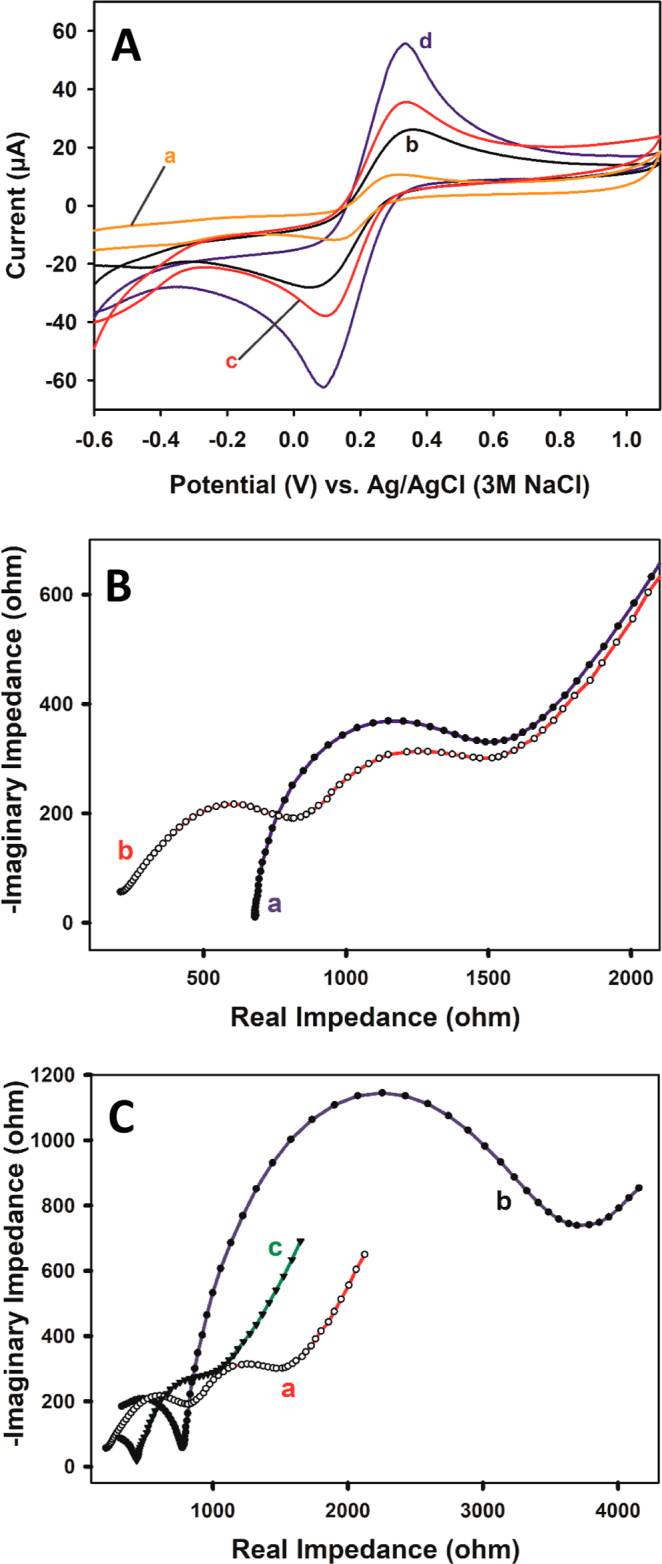
(A) Cyclic voltammograms of (a) bare PGE, (b) aPGE, (c) pTRT/PGE, and (d) pTRT/aPGE with a scan rate of 50 mV/s, (B) EIS spectra of (a) aPGE and (b) pTRT/aPGE and (C) electro-polymerized tartrazine electrodes onto aPGE in the solutions of (a) 0.1 M pH 5 acetic acid/acetate buffer solution, (b) 0.1 M pH 7 PBS, and (c) 0.1 M NaOH within the frequency range of 0.1–100,000 Hz in the presence of 1 mM of K_3_[Fe(CN)_6_], 1 mM of K_4_[Fe(CN)_6_], and 0.1 M of KCl. The single points represent the experimental results, and the lines represent the fitted curves.

Characterization measurements for the produced platforms, i.e., pTRT/aPGE and the other related platforms, were performed by various techniques involving CV, EIS, SEM, EDX, and XPS. When CV measurements were recorded in the presence of 1 mM of K_3_[Fe(CN)_6_], 1 mM of K_4_[Fe(CN)_6_], and 0.1 M of KCl (Figure 2A), the lowest current was obtained from the bare PGE [Figure 2A(a)], and the peak current values consecutively increased with activated PGE (aPGE) [Figure 2A(b)], poly(tartrazine)-modified PGE (pTRT/PGE) [Figure 2A(c)], and poly(tartrazine)-modified aPGE (pTRT/aPGE) [Figure 2A(d)]. The fact that the peak current was higher than aPGE [Figure 2A(b)] even in pTRT/PGE [Figure 2A(c)] indicated that the polymer film had a catalytic effect. With pTRT modified onto the aPGE, it was observed that the current increased by 420% in pTRT/aPGE compared to bare PGE.

The EIS spectra of aPGE and pTRT/aPGE are demonstrated in Figure 2B, while EIS spectra of the pTRT/aPGE electro-polymerized in pH 5 acetic acid/acetate buffer solution, pH 7 PBS, and NaOH solution appear in Figure 2C.

EIS data were fit with different circuits of *R*_s_(*C*_dl_[*R*_ct_*W*]) for aPGE, [*R*_s_(CPE_f_*R*_f_)(CPE_dl_*R*_ct_)*W*] for pTRT/aPGE electro-polymerized in pH 5 acetic acid/acetate buffer solution, and [*R*_s_(CPE_h_[*R*_h_(CPE_f_*R*_f_)(CPE_dl_*R*_ct_)])] for pTRT/aPGE electro-polymerized in pH 7 PBS and NaOH solution (Table S2). *R*_s_ is the resistance from solution, electrode, wires, and connectors, *R*_ct_ is the charge-transfer resistance, *C*_dl_ and CPE_dl_ are the capacitance of the double-layer, *W* is Warburg impedance; *R*_h_ and CPE_h_ are the resistance and capacitance of the incomplete capacitive semi-circle at the high-frequency region, and *R*_f_ and CPE_f_ are the resistance and capacitance of the polymer film.^41^ A well-known circuit, *R*_s_(*C*_dl_[*R*_ct_*W*]), consists of a semi-circle corresponding to *R*_s_(*C*_dl_*R*_ct_) at a high-frequency region and a linear region related to Warburg impedance (*W*) at a low-frequency region for aPGE.^28^ Another semi-circle emerged at a high-frequency region for pTRT/aPGE electro-polymerized in pH 5 acetic acid/acetate buffer solution shows a uniform polymer film layer where current must first flow through this layer and then the inner part of the electrode (i.e., graphite surface).^42^ Therefore, circuit (CPE_f_*R*_f_), representing the pTRT film, is added to the *R*_s_(*C*_dl_[*R*_ct_*W*]) circuit for aPGE. After the electro-polymerization of tartrazine in the presence of pH 7 PBS or NaOH solution, the incomplete capacitive semi-circles at a high-frequency region arise due to the irregularity of the current flow at the formed polymer film layer.^41^ Therefore, in addition to the (CPE_f_*R*_f_) circuit representing the polymer film, the (CPE_h_*R*_h_) circuit is added owing to the current flow abnormality. In these circuits, the reason for removing the *W* is that the tangent angle of the linear part differs from 45° due to the current flow irregularity. Therefore, when the *W* is added to the circuit, a fitted-curve compatible with the experimental data cannot be obtained. These abnormalities are due to the disruption of coordination between the azo group and the adjacent hydroxyl group in the tartrazine molecule at neutral and basic pH values. At acidic pH values between 4.2 and 6.5, polymerization takes place uniformly since the azo group and the adjacent hydroxyl group are in coordination.^43,44^ All EIS data and findings were found to be compatible with the literature.^28,41,42^

The conductivity values of each polymer film-modified electrode greatly increase compared to aPGE from the obtained *R*_s_ values. Besides, due to the non-uniformity of the polymer, the resistance of the polymer film (*R*_f_) is higher in the coating carried out in NaOH than that in the presence of pH 7 PBS. This is due to the presence of tartrazine in the anionic form in the basic solution and the partial anionic character in the resulting polymer. Therefore, the *R*_f_ value is greater due to the repulsive force between this anionic polymer and the redox pair [Fe(CN)_6_^3–^/Fe(CN)_6_^4–^].^41,44^

In addition, *W* values show that pTRT/aPGE is more efficient in diffusion-induced substance transport than aPGE, as listed in Table S2.

SEM images for bare PGE, aPGE, and pTRT/aPGE appear in Figure S1. Similar to our previous study,^45^ the bare PGE does not show a uniform distribution (Figure S1A), while it becomes more uniform and has a channeled structure when activated (Figure S1B). As expected, these channels fill with polymer (i.e., pTRT) when the TRT is coated onto the aPGE (Figure S1C).

EDX spectra of the bare PGE, aPGE, and pTRT/aPGE appear in Figure S2. It is observed that bare PGE contains carbon (98.1%) and oxygen (1.9%),^41,45^ and after activation, the oxygen ratio (15.7%) increases considerably.^45^ With the electro-polymerization of TRT, nitrogen (4.0%) due to the azo group in the TRT structure, a small amount of sulfur (0.2%) due to the sulfo groups of TRT, and a small amount of sodium (0.1%) possibly diffused into the polymer structure due to the salt form of TRT are observed in the EDX spectrum.

**Figure 3:**
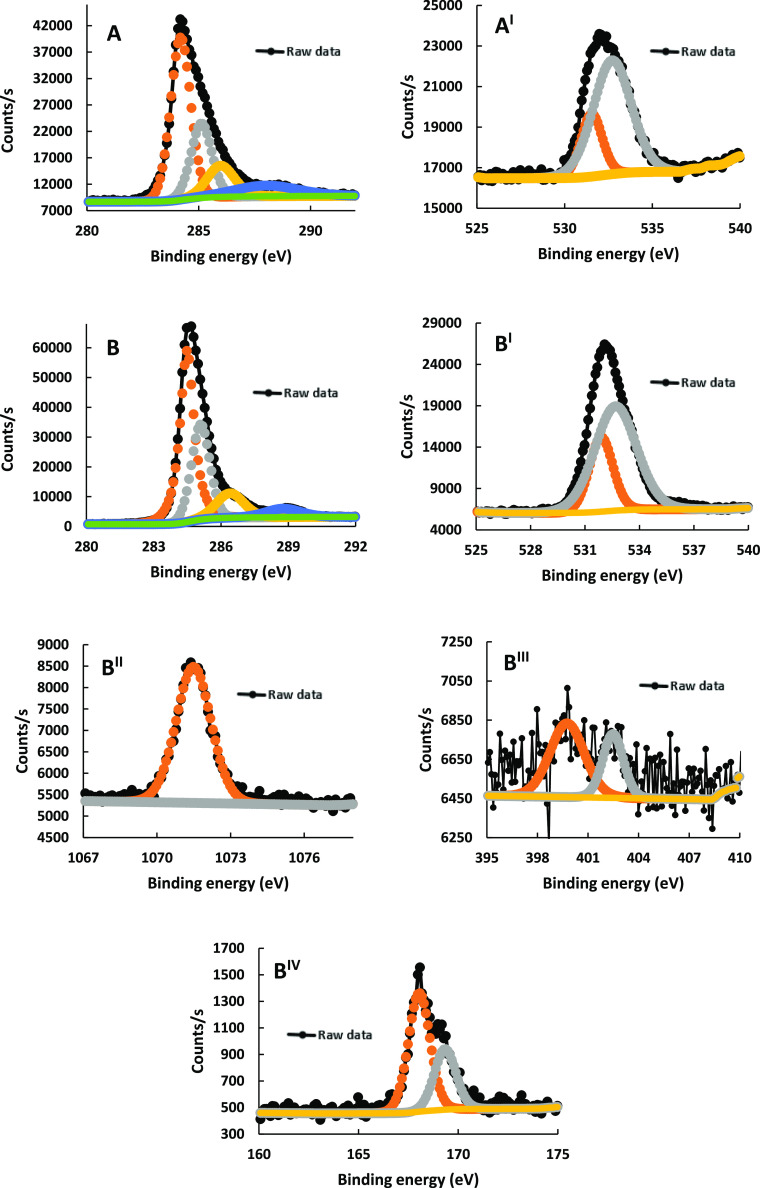
XPS spectra for aPGE (A,A^I^) and pTRT/aPGE (B,B^I^,B^II^,B^III^,B^IV^). XPS analysis: Al Kα gun, 300 μm spot size, 50 eV pass energy, and 0.1 eV energy step size. (A,B) Carbon 1s, (A^I^,B^I^) oxygen 1s, (B^II^) sodium 1s, (B^III^) nitrogen 1s, and (B^IV^) sulfur 2p. The black dotted lines are raw data, and the others are fitted curves.

XPS measurements were performed to further explain the electro-polymerization that occurred onto the aPGE, as shown in Figure 3. The formal oxidation states between 284.3 and 286.6 eV correspond to the C–C and C–O–C structures, and the formal oxidation states at 288.2 and 289.2 eV belong to the C=O groups (Figure 3A,B).^43,45^ An approximately two-fold increase in the C 1s signal at pTRT/aPGE obviously signifies a carbon-based formation on the surface (i.e., polymer of TRT).^45^ The BEs at 531.6 and 531.8 eV belong to the C=O groups, and the BEs of O 1S at 532.9 and 533.1 eV are due to the partial aromatic C–O–C structures of graphite^46^ and/or organic C–O–C structures of the obtained polymer,^47^ respectively (Figure 3A^I^,B^I^).

The formal oxidation state at 1071.6 eV may be attributed to the diffused sodium ions into the pTRT from the salt of TRT (Figure 3B^II^).^45^ The two most important pieces of evidence of electro-polymerization are the emergence of N 1S and S 2p chemical environments in XPS measurements.^47,48^ N 1S spectra show the presence of −N–C structures at 399.7 eV and imine (−C=N) structures at 402.5 eV (Figure 3B^III^).^49^ The BEs of S 2p_1/2_ and S 2p_3/2_ at 169.5 and 168.2 eV correspond to the SO_3_ structures (Figure 3B^IV^).^50^

As a consequence, greatly harmonic characterization data obtained from CV, EIS, SEM, EDX, and XPS measurements demonstrate that the pTRT/aPGE is properly manufactured for determining the UA in synthetic and real samples.

Considering the characterization data and the oxidation reaction of TRT, the possible electro-polymerization mechanism of TRT and the voltammetric oxidation of UA are proposed in accordance with the literature, as depicted in Figure 1A.^51−53^

### 3.2 Cyclic Voltammetric Characteristics of the System

The Randles–Sevcik equation is used to calculate the effective surface areas of aPGE and pTRT/aPGE from the slope (*I*_p_ – *v*^1/2^) of the equation
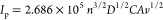
where *I*_p_, *n*, *D*, *C*, *A*, and *v* refer to the peak current (ampere), the number of transferred electrons, the diffusion coefficient (cm^2^/s), and the concentration (mol/cm^3^) of the redox couple, the effective surface area (cm^2^) and the scan rate (V/s), respectively. Of these, *n* and *D* are 1 and 7.6 × 10^–6^ cm^2^/s for the redox couple ([Fe(CN)_6_]^3–^/[Fe(CN)_6_]^4–^), respectively. The effective surface areas were found as 15.5 mm^2^ for aPGE and 33.4 mm^2^ for pTRT/aPGE, depicting a 115% increase.

The Brown–Anson equation is applied to reckon the surface coverage of pTRT layer from the slope (*I*_p_ – *v*) of the equation
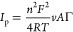
where *F*, *R*, *T*, and Γ are the Faraday constant (96,485 Coulomb/mol), the gas constant (8.314 J/mol·K), temperature (K), and the surface coverage (mol/cm^2^), respectively, and it was found as 4.52 nmol/cm^2^.

The electrode reaction mechanism belonging to the translocation of UA was examined by recording CV voltammograms at increasing scan rates varied from 10 to 1000 mV/s (Figure S3B). The logarithm of peak height (log(*I*_p_, μA)) was plotted against the logarithm of the scan rate (log(*v*, mV/s)), and the related equation with a slope of 0.788, log(*I*_p_) = 0.788 log(*v*) + 0.515 (*R*^2^: 0.994), depicted that the electrode reaction was on the basis of a joint diffusion- and adsorption-controlled system (Figure S3C). The reason why adsorption accompanies diffusion is due to the fact that UA, an organic substance, tends to be close to the pTRT/aPGE, which has also an organic structure.^54^

The apparent charge-transfer coefficient (α) was calculated from the slope (2.3*RT*/[(1 – α)*nF*]) of the *E*_p_(mV) – log(*v*, mV/s) curve, *E*_p_ = 81.505 log(*v*) + 147.340 (*R*^2^: 0.984), as 0.64 (Figure S4). It is concluded that the transition state acts asymmetrically between the responses of UA and UA-4,5-diol against the applied potential.^55,56^

The slope of the *E*_p_ – pH curve, −64 mV/pH, shows that the ratio of proton to electron occurring at the surface of pTRT/aPGE is 1, which is known to correspond to 2-electron and 2-proton for UA oxidation (Figure S5).^51^

**Figure 4:**
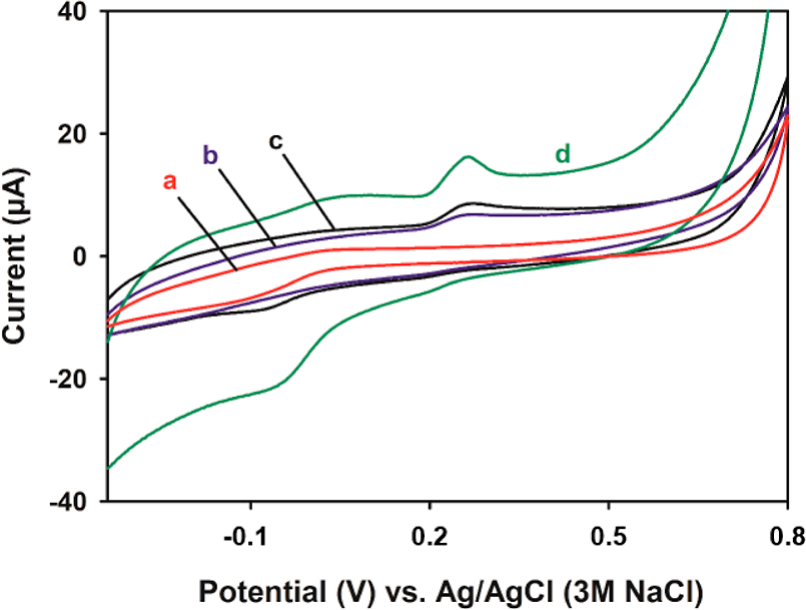
Cyclic voltammograms of (a) bare PGE, (b) aPGE, and (c) pTRT/PGE and (d) pTRT/aPGE in the presence of UA. Conditions: 50 μM of UA, 0.05 M (pH 7.5) of PBS solution, *E*_start_: −1 V, *E*_first_: 1 V, *E*_finish_: −1 V, step amplitude: 3 mV, and scan rate: 50 mV/s.

Furthermore, CV measurements were performed with bare PGE, aPGE, pTRT/PGE, and pTRT/aPGE in a solution containing 50 μM of UA to exhibit the electrocatalytic effect of the produced platform, as shown in Figure 4. No oxidation peak is observed at bare PGE for UA, while the related peak currents at aPGE, pTRT/PGE, and pTRT/aPGE are 6.96, 8.82, and 16.04 μA, respectively. The results show the current rising about 26.7% for pTRT/PGE over bare aPGE, about 81.9% for pTRT/aPGE over pTRT/PGE, and about 130% for pTRT/aPGE over aPGE. Consequently, the results demonstrate that the pTRT film causes an electrocatalytic effect on UA oxidation similar to our previous study for quercetin oxidation.^45^

### 3.3 Parameters Affecting the UA Determination

Significant parameters for the electro-polymerization involving pencil graphite grade, the concentration of TRT and pH 5 acetic acid-acetate buffer solution, scan rate, and the number of CV cycles, and for the analysis including pH and the concentration of PBS solution and ionic strength were investigated with pTRT/aPGE using 30 μM of UA in the relevant ranges by plotting the peak height (*I*_ph_) against the examined parameter and determined to be 2B, 0.5 mM, 0.1 M, 100 mV/s, 15, 7.5, 0.05 M, and 0, as appeared in Figure S6, respectively.

### 3.4 Method Validation

**Figure 5:**
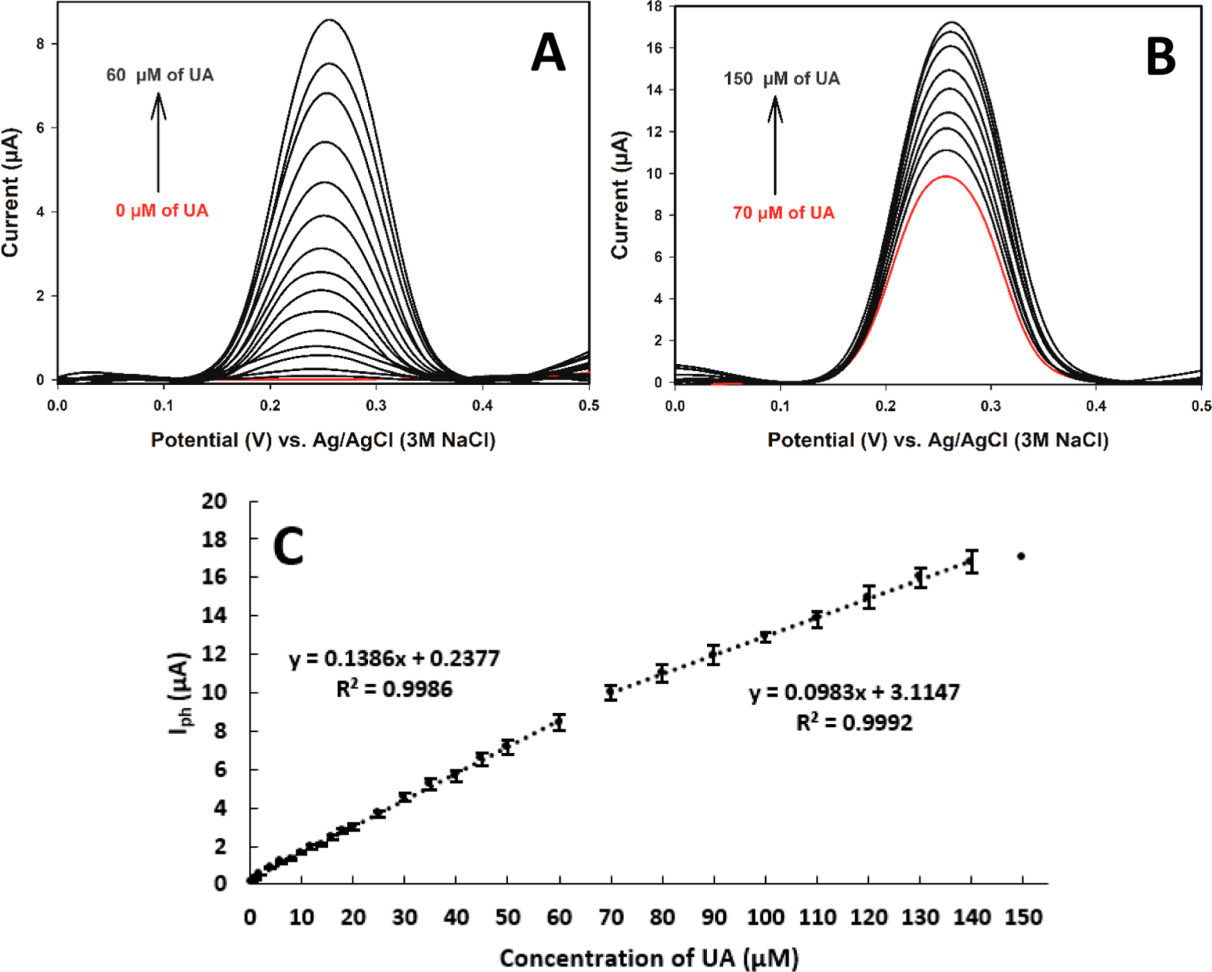
(A,B) DPV voltammograms and (C) calibration curves belong to the UA in 0.05 M (pH 7.5) of PBS solution (*n* = 3 for each concentration). Conditions: *E*_start_: −0.4 V, *E*_finish_: 1.0 V, step amplitude: 5 mV, pulse amplitude: 25 mV, and scan rate: 25 mV/s.

The DPV results and calibration curves of electrochemical UA oxidation are shown in Figure 5. With the oxidation of the unsaturated carbon bond in the middle of the purine ring, UA-4,5-diol is formed, and the peak height increases commensurately with UA.^57^ The significant analytical parameters including LOD and linear ranges for UA detection are 0.10 μM [i.e., from the blank signal, LOD = 3 *s*/*m*, where *s* is the standard deviation of the blank solutions (*n* = 6) and *m* is the slope of the calibration curve], 0.34–60, and 70–140 μM, respectively. The sensitivity of the polymer film-modified electrode was obtained as 0.415 μA·μM^–1^·cm^–2^ depicting a remarkably sensitive platform for UA detection. Furthermore, the chronoamperomograms and calibration curve of amperometric measurements appear in Figure S7.

The reproducibility and repeatability of the produced platform, pTRT/aPGE, were investigated at 10, 30, 60, and 100 μM UA, and the relative standard deviation (RSD %) values were found to be 2.7–5.1 and 1.3–3.3%, respectively (the number of replicates: 6). The results show that the produced platform has remarkable reproducibility and repeatability.

The stability of the pTRT/aPGE was investigated in UA-spiked samples as well as 12 d following the first measurement. The peak heights decreased by 7.2 and 4.6% for solutions containing 10 and 90 μM of UA, respectively. Those results indicate the exceptional stability of pTRT/aPGE as well.

Interference effects of dopamine, ascorbic acid, urea, glucose, sucrose, potassium chloride, sodium chloride, sodium sulfate, sodium carbonate, magnesium chloride, calcium chloride, and sodium nitrate were examined in the presence of 20 μM UA using pTRT/aPGE based on a criterion of ±5% change in peak height, as detailed in Table S3. From the results obtained, it was found that even dopamine, which has the most effect on UA determination, changed the peak height by less than 5% at 40 μM. Accordingly, the results show that the produced sensor works selectively for determining UA.

### 3.5 Sample Application

Human serum and artificial human urine samples were analyzed by both the developed method and the amperometric method. The voltammograms, chronoamperomograms, and the results for these samples are given in Figures S8 and S9 and Table 1, respectively. The recovery and RSD % values for artificial human urine samples by the proposed and the amperometric method are 206.46 ± 9.77 and 194.01 ± 15.73 μM, respectively. The UA content in human serum was found to be 288.29 ± 7.06 μM with the developed method and 288.42 ± 15.84 μM with the amperometric method. The UA amounts calculated by multiplying the results obtained from both methods with the dilution factors were found to be compatible with the literature, i.e., 208.2–428.3 μM.^58^

**Table 1:** Sample Application Results (*n* = 6)^a^

samples	voltammetric UA determination^b^			amperometric UA determination^b^				
	UA found (μM)	recovery (%)	relative standard deviation (%)	UA found (μM)	recovery (%)	relative standard deviation (%)	*F*_experimental_^c^	*t*_experimental_^d^
artificial human urine	206.46 ± 9.77	103.23 ± 4.88	4.73	194.01 ± 15.73	97.00 ± 7.87	8.11	2.60	1.65
human serum	288.29 ± 7.06		2.45	288.42 ± 15.84		5.49	5.03	0.02

a Conditions: Real samples in 0.05 M (pH 7.5) of PBS solution, *E*_start_: −0.4 V, *E*_finish_: 1.0 V, step amplitude: 5 mV, pulse amplitude: 25 mV, and scan rate: 25 mV/s.

b All results are obtained by multiplying the relevant dilution factors stated in Section 2.5.

c *F*_critical_ is 5.050 for 5; 5 degrees of freedom.

d *t*_critical_ is 2.228 for 10 degrees of freedom.

In addition to the aforementioned measurements, standard addition of UA was applied to both human serum and artificial human urine samples at various concentration levels. The accuracy of the method was further evaluated through recovery values, while precision was assessed using RSD % values. According to the data presented in Table S4, the measured levels of UA exhibit strong concordance with the spiked concentrations, demonstrating high detection recoveries ranging from 96.76 to 104.76%. The repeated electrochemical DPV measurements show low RSD values and a narrow distribution ranging from 1.44 to 4.36%. These findings affirm the high reliability and suitability of using pTRT/aPGE for UA detection in both human serum and artificial human urine samples, with a substantial agreement between measured and spiked UA concentrations, along with notable detection recovery and low RSD.

In order to interpret the precision and trueness of the proposed method, the results were evaluated statistically by comparing the amperometric and the suggested method by applying *F*-test and *t*-test. The fact that the experimental *F* (*F*_experimental_) and *t* (*t*_experimental_) values are smaller than the critical *F* (*F*_critical_) and *t* (*t*_critical_) values indicates that the developed method gives an accurate response at the 95% confidence interval (Table 1).

## 4 Conclusions

A unique, disposable, and cheap electrochemical sensor based on a polymer film of tartrazine prepared in a single step was designed and developed for determining UA in human serum and artificial human urine samples. The synthesized polymer film was thoroughly characterized by numerous techniques such as CV, EIS, SEM, EDX, and XPS. Collectively, the increase in the effective surface area of the produced electrode, the improvement of the electron conduction rate and therefore the conductivity, and the formation of a uniform polymer layer exhibited an electrocatalytic oxidation of UA by raising the peak height of about 130% over bare PGE.

The produced platform, pTRT/aPGE, reached an outstanding LOD and sensitivity, and wide linear ranges as 0.10 μM, 0.415 μA·μM^–1^·cm^–2^, 0.34–60, and 70–140 μM, respectively. Furthermore, the analytical performance of the pTRT/aPGE was enhanced with sample application of human serum and artificial human urine. The results of the proposed sensor were compared to the results of the amperometric method, and the significant statistical difference between the methods was not found at 95% confidence interval implying the good accuracy of the developed method.

## Supporting Information Available

Electrochemical methods for determination of UA; CV, EIS, SEM, and EDX measurements; parameters affecting the UA determination; chronoamperometric measurements; interference studies; and results of sample application (PDF)
