# Supplementary material for: Electrocatalytic Determination of Uric Acid with the Poly(Tartrazine)-Modified Pencil Graphite Electrode in Human Serum and Artificial Urine
Source: ACS Omega. 2023 Sep 11;8(38):34420–30. doi: 10.1021/acsomega.3c02561 (PMC10535258; doi:10.1021/acsomega.3c02561)
Supplement: Supplementary file 1 — ao3c02561_si_001.pdf [file ao3c02561_si_001.pdf]

Electrocatalytic Determination of Uric Acid with the Poly(Tartrazine)-Modified Pencil Graphite Electrode in Human Serum and Artificial Urine

Lokman Liv[a*], Merve Portakal[a,b], Meryem Sıla Çukur[a,c], Beyza Topaçlı[a,d], Berkay Uzun[a,c]

[a]Electrochemistry Laboratory, Chemistry Group, The Scientific and Technological Research Council of Turkey, National Metrology Institute, (TUBITAK UME), 41470, Gebze, Kocaeli, Turkey.

[b]Pamukkale University, Faculty of Technology, Department of Biomedical Engineering, 20160, Denizli, Turkey.

[c]Kocaeli University, Faculty of Technology, Department of Biomedical Engineering, 41380, Kocaeli, Turkey.

[d]TOBB University of Economics and Technology, School of Engineering, Department of Biomedical Engineering, 06560, Ankara, Turkey.

E-mail: lokman.liv@tubitak.gov.tr

Contents **Page**

1. Introduction…………………………………………………………………………………………………S2

2. Surface characterization of pTRT/aPGE…………………………………………………………………S3

3. Cyclic voltammetric characteristics of the system…………………………………………………S5

4. Parameters affecting the UA determination ……………………………………………………….S7

5. Method validation……………………………………………………………………………………………S8

6. Sample application……………………………………………………………………………………………S9

1. Introduction

Table S1. Electrochemical methods for determination of uric acid.

Sensing platform	Method	LOD (µM)	Analytical range (µM)	Sample application	Reference
Methylcellulose/graphene oxide/iron oxide nano hydrogel/glassy carbon electrode	Differential pulse voltammetry	0.17	0.5–140	Human urine	1
β-cyclodextrin/reduced graphene oxide/screen printed electrode	Differential pulse voltammetry	0.026	0.08–150	Human serum	2
(Platinum nanoparticles)-graphene flakes-flavin mononucleotide/gold interdigitated microelectrode	Cyclic voltammetry	18	60–284, 60–578	-	3
Multiwalled carbon nanotubes/ poly(4-amino-3-hydroxy naphthalene sulfonic acid)/glassy carbon electrode	Square wave voltammetry	0.024	1–100	Human urine	4
Acrylic acid-ethylene glycol dimethacrylate-2,2'-azobis(2-isobutyro) nitrile/carbon paste electrode	Differential pulse adsorptive stripping voltammetry	0.1	0.5–100	Human serum	5
Nickel ferrite/glassy carbon electrode	Differential pulse voltammetry	0.15	0.398–6.761	Human urine	6
Nickel hydroxide/solar graphene/glassy carbon electrode	Differential pulse voltammetry	0.46	2–15	Human serum and urine	24
Platinum nanoparticles/multiwalled carbon nanotubes/glassy carbon electrode	Differential pulse voltammetry	0.35	0.45–50	-	25
Copper nanoparticles/polypyrrole/glassy carbon electrode	Differential pulse voltammetry	0.0008	0.001–10	Human urine	26
Poly(p-aminophenol)/glassy carbon electrode	Differential pulse voltammetry	0.25	0.4–150	-	27
Platinum nanosheets/fullerene/glassy carbon electrode	Differential pulse voltammetry	0.63	9.5–1187	Human serum and urine	17
Nickel ferrite nanorods/sulfur doped carbon nanoparticles/glassy carbon electrode	Differential pulse voltammetry	0.017	0.3–30	Human urine	18
Poly(solid red a)/carbon nanotube paste electrode	Cyclic voltammetry	1	2–64	Human urine	19

N,P-doped hollow mesoporous carbon nanospheres-phytic acid/glassy carbon electrode	Differential pulse voltammetry	0.005	0.5–5000	Human serum and urine	20
SnO_2/graphene/glassy carbon electrode	Square wave voltammetry	0.28	0.1–200	Human urine	21
Three-dimensional porous graphene/glassy carbon electrode	Differential pulse voltammetry	1	1–60	-	22
Poly(glyoxal-bis(2-hydroxyanil))/ glassy carbon electrode	Differential pulse voltammetry	0.09	1–100	Human urine	23
Gold clusters/N-acetyl-L-cysteine–multiwalled carbon nanotubes/ glassy carbon electrode	Differential pulse voltammetry	0.04	0.1–300	Human serum and urine	29
Gold-silver bimetallic nanoparticles/graphene oxide/thionine/glassy carbon electrode	Square wave voltammetry	0.3	1–100	Human serum and urine	30
Urate oxidase/metal organic framework (gold nanocage)/glassy carbon electrode	Square wave voltammetry	0.015	0.05–55	Human serum and urine	31
Urate oxidase-cobalt-based metal-organic framework/boron nanosheets-doxorubicin/glassy carbon electrode	Square wave voltammetry	0.025	0.1–200	Human serum and urine	32
Poly(tartrazine)/pencil graphite electrode	Differential pulse voltammetry	0.10	0.34–60 µM, 70–140 µM	Human serum and artificial human urine	This work

2. Surface characterization of pTRT/aPGE

Table S2. Produced platforms and the equivalent circuits obtained from EIS data.

Electrode	Equivalent circuit	Error (%)
aPGE	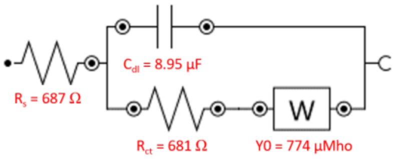	3.0
pTRT/aPGE (electro-polymerized in pH 5 acetic acid/acetate buffer solution)	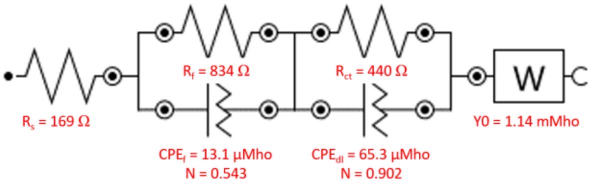	2.8

pTRT/aPGE (electro-polymerized in pH 7 phosphate buffer solution)	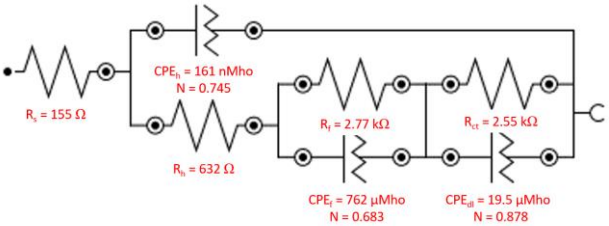	0.8
pTRT/aPGE (electro-polymerized in NaOH solution)	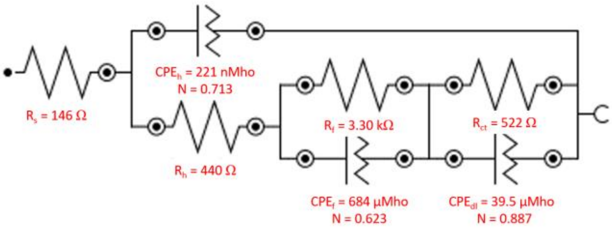	0.4

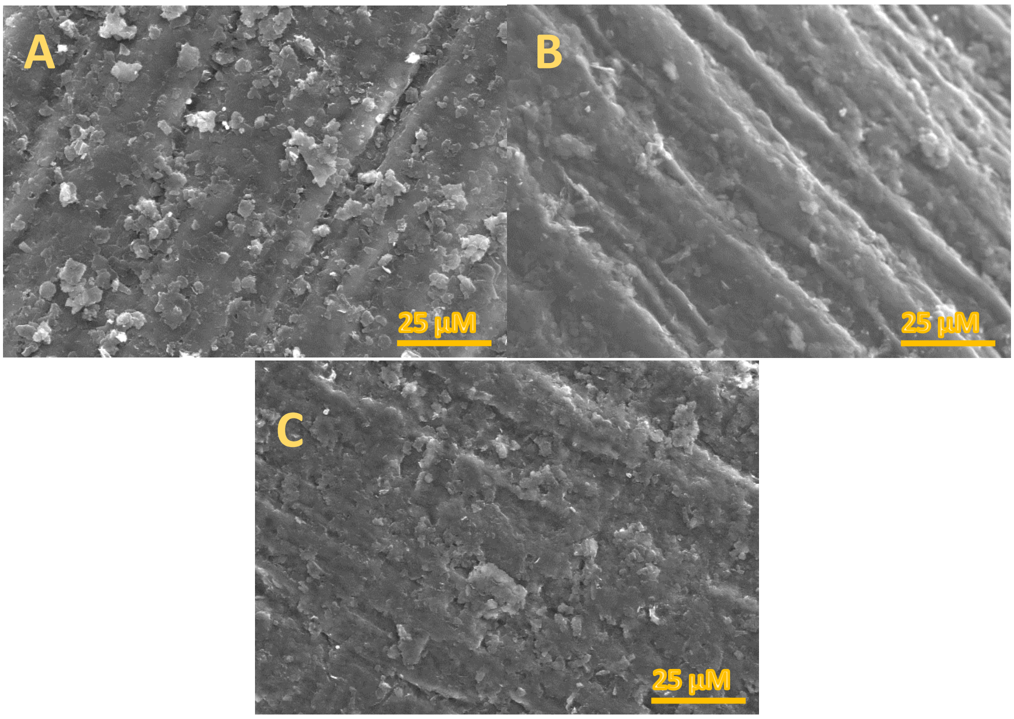

Figure S1. SEM images of (A) bare PGE, (B) aPGE and (C) pTRT/aPGE. (SEM analysis: 15 kV voltage, 30 spot intensity, SE(L) detector).

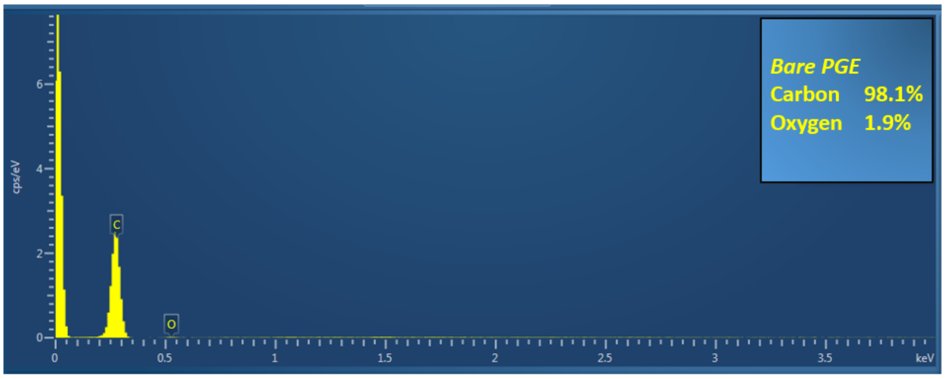

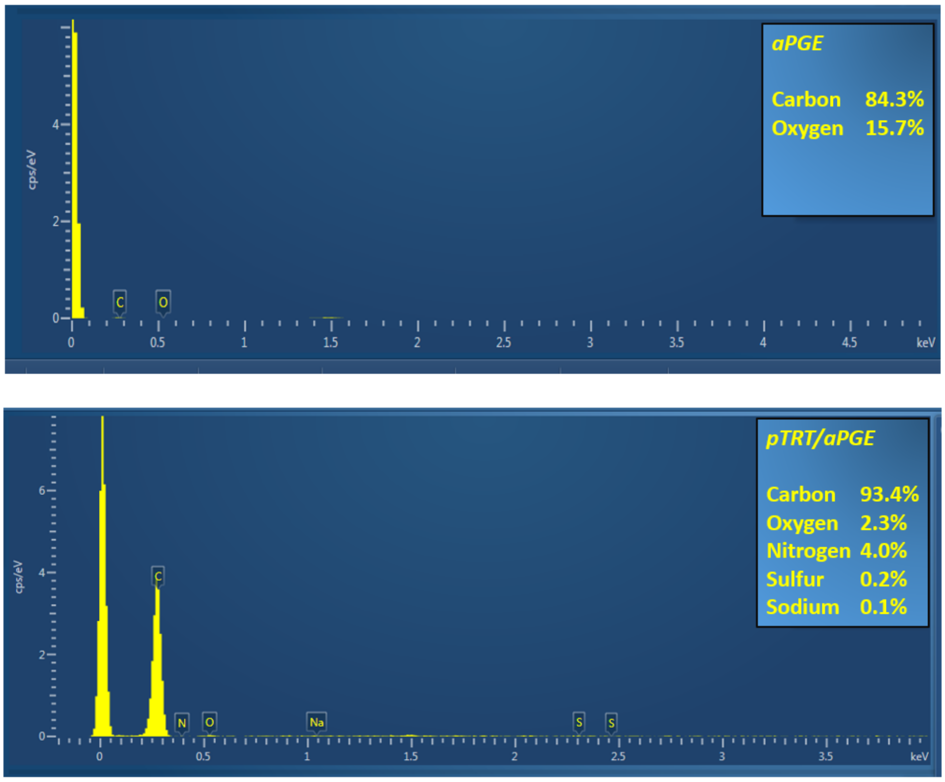

Figure S2. EDX spectra for bare PGE, aPGE and pTRT/aPGE. (EDX analysis: 15 kV, 100 µm × 100 µm, AZtec software, mass percentages were given in EDX spectra).

3. Cyclic voltammetric characteristics of the system

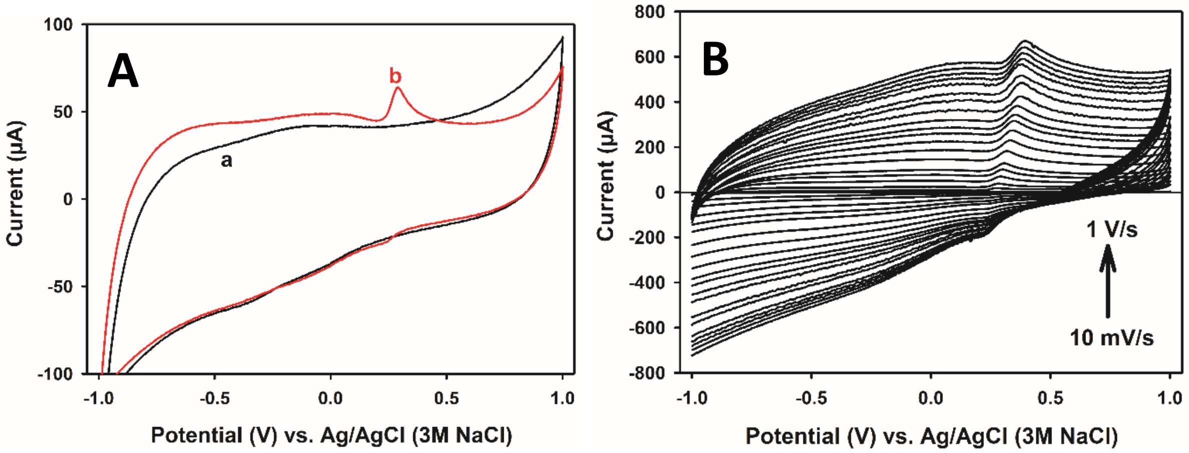

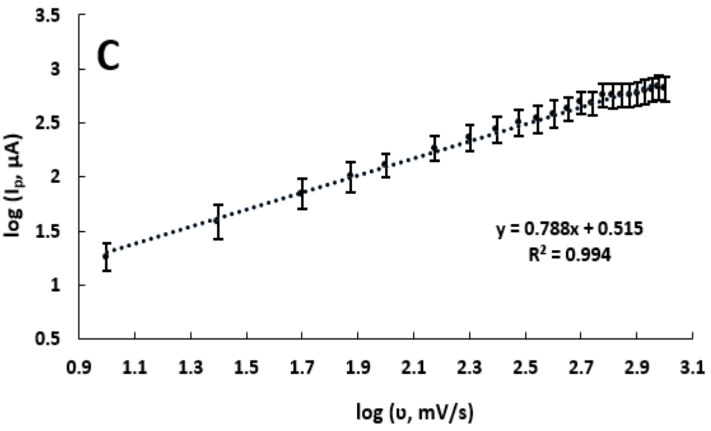

Figure S3. (A) Cyclic voltammograms of pTRT/aPGE at 50 mV/s (a) in the absence and (b) the presence of UA, (B) cyclic voltammograms of UA at increasing scan rates using pTRT/aPGE and (C) $\log(I_p) - log(v)$ curve. Conditions: 100 µM of UA, 0.05 M (pH 7.5) of PBS solution, E_{start}: -1 V, E_{first}: 1 V, E_{finish}: -1 V, step amplitude: 3 mV.

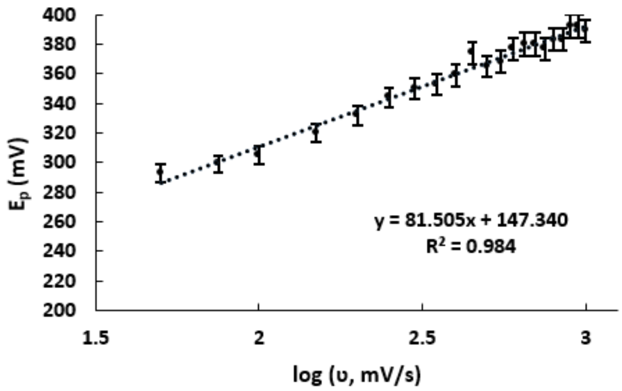

Figure S4. $E_p - log(v)$ curve obtained with pTRT/aPGE. Conditions: 100 µM of UA, 0.05 M (pH 7.5) of PBS solution, E_{start}: -1 V, E_{first}: 1 V, E_{finish}: -1 V, step amplitude: 3 mV.

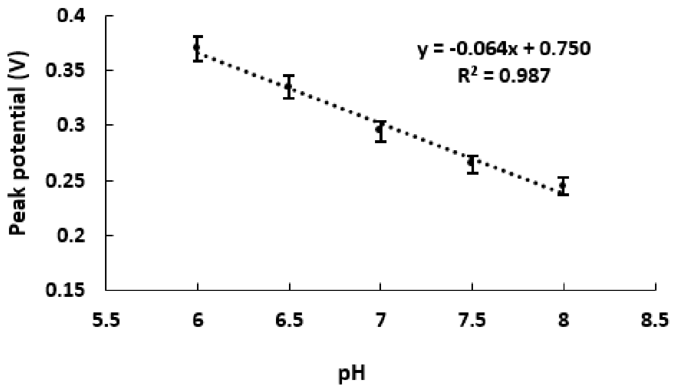

Figure S5. $E_p - pH$ curve obtained with pTRT/aPGE. Conditions: 30 µM of UA, 0.05 M of PBS solution at different pH values, E_{start}: -0.4 V, E_{finish}: 1.0 V, step amplitude: 5 mV, pulse amplitude: 25 mV, scan rate: 25 mV/s.

4. Parameters affecting the UA determination

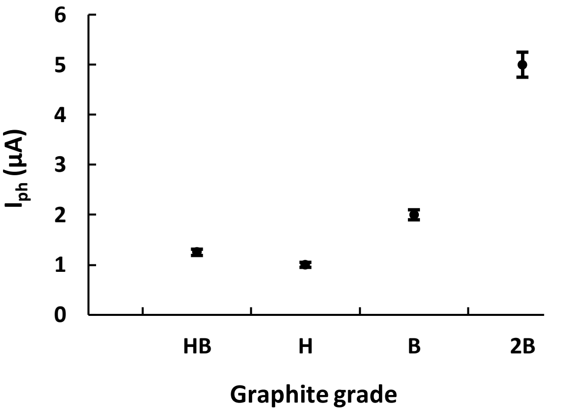

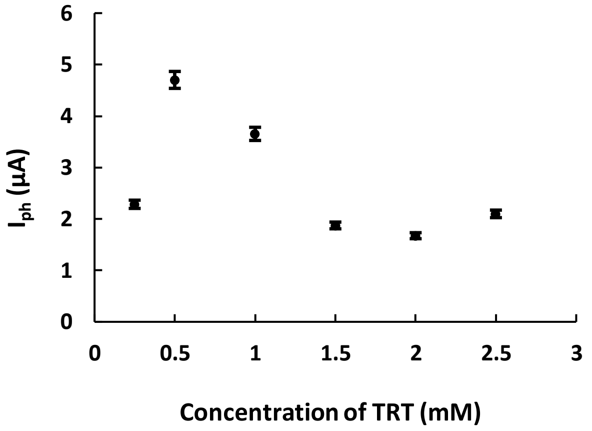

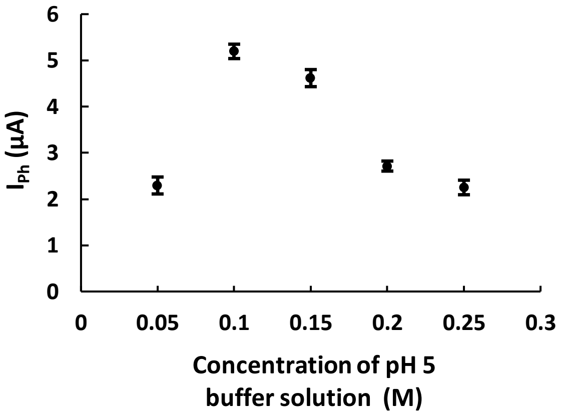

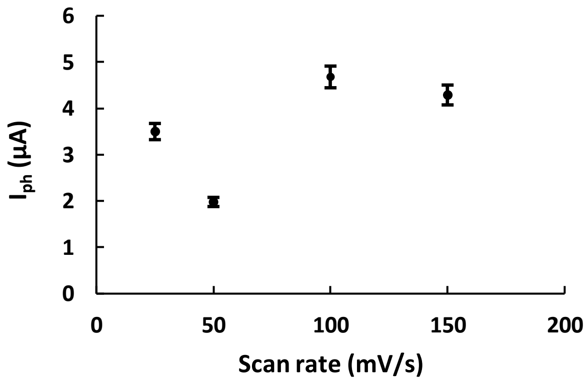

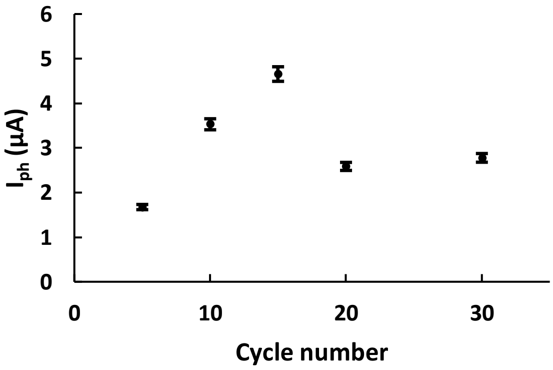

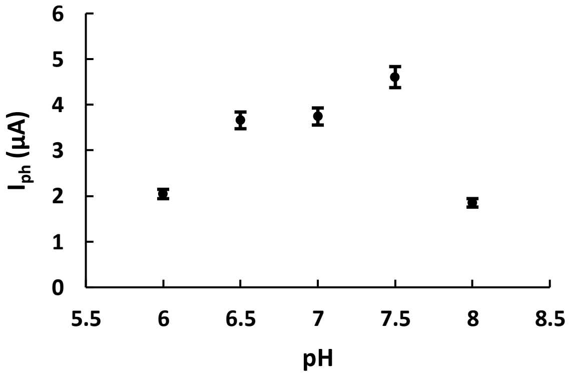

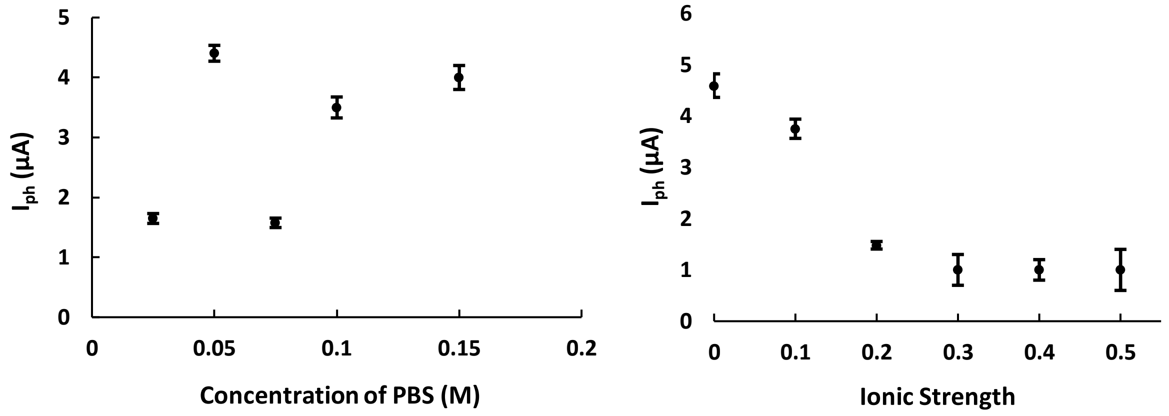

Figure S6. The parameters affecting the electro-polymerization including pencil graphite grade, the concentration of TRT and pH 5 acetic acid-acetate buffer solution, scan rate and the number of CV cycles, and for the analysis including pH and the concentration of PBS solution and ionic strength. Conditions: 30 µM of UA. E_{start}: -0.4 V, E_{finish}: 1.0 V, step amplitude: 5 mV, pulse amplitude: 25 mV, scan rate: 25 mV/s.

5. Method validation

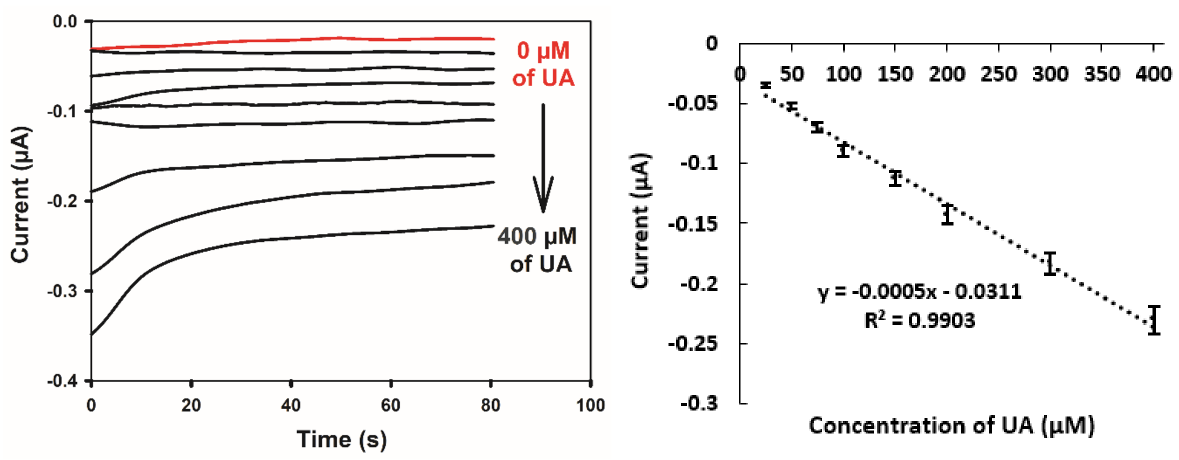

Figure S7. Chronoamperomograms and calibration curve belong to the UA in 0.02 M (pH 8) of borate buffer solution (n= 3 for each concentration). Conditions: Set potential: -100 mV, interval time: 0.5 s.

Table S3. Interference studies and tolerable amounts of various compounds in the presence of 20 µM UA with pTRT/aPGE. Conditions: 0.05 M of pH 7.5 PBS. E_{start}: -0.4 V, E_{finish}: 1.0 V, step amplitude: 5 mV, pulse amplitude: 25 mV, scan rate: 25 mV/s.

Interferences	Tolerable amount (µM)
Sodium chloride, potassium chloride	2000
Sodium sulfate	1000
Sucrose, glucose	500
Urea	300
Glycine, L-cysteine	240
Magnesium chloride	220
Sodium carbonate	200
Calcium chloride, sodium nitrate	140
Ascorbic acid	120
Citric acid	80
Dopamine	40

6. Sample application

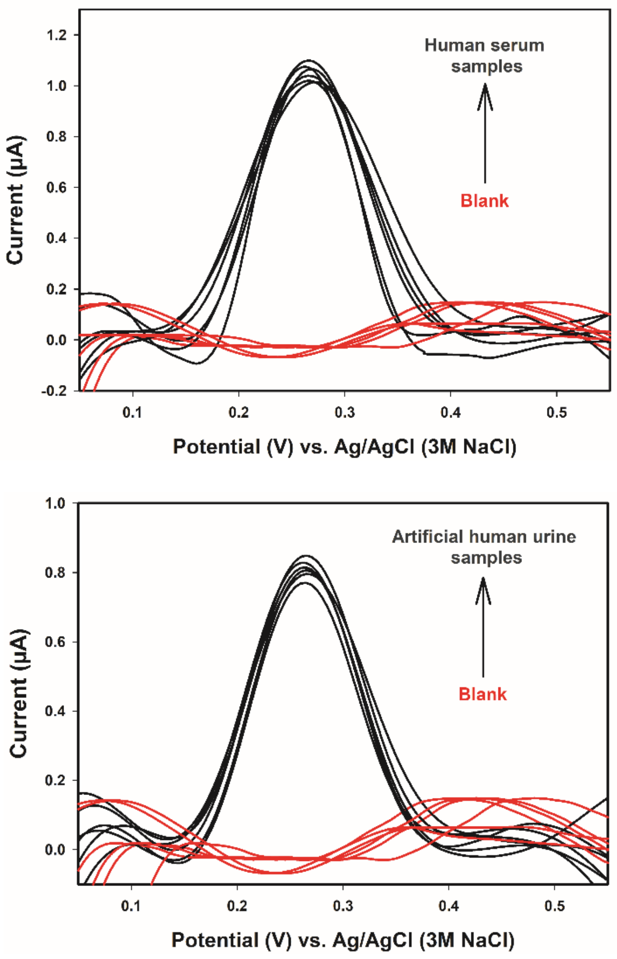

Figure S8. DPV voltammograms belong to the samples of human serum and artificial human urine. Conditions: 50-fold diluted amounts of real samples in 0.05 M (pH 7.5) of PBS solution. E_{start}: -0.4 V, E_{finish}: 1.0 V, step amplitude: 5 mV, pulse amplitude: 25 mV, scan rate: 25 mV/s.

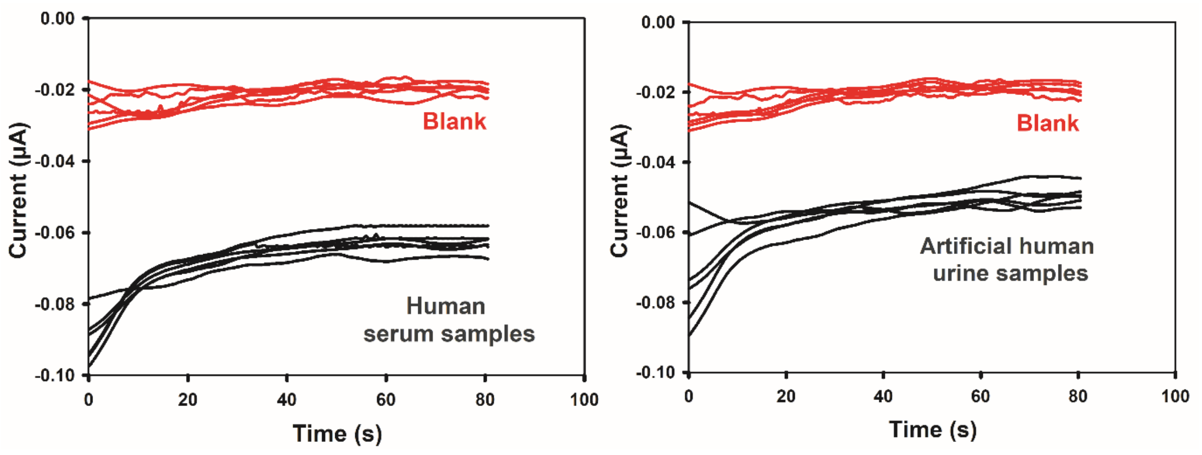

Figure S9. Chronoamperomograms belong to the samples of human serum and artificial human urine with a commercial uricase modified screen printed electrode. Conditions: 4-fold diluted amounts of real samples in 0.02 M (pH 8) borate buffer solution. Set potential: -100 mV, interval time: 0.5 s.

Table S4. Recovery measurement results (n=6) of human serum and artificial human urine samples. Conditions: Real samples in 0.05 M (pH 7.5) of PBS solution, E_{start}: -0.4 V, E_{finish}: 1.0 V, step amplitude: 5 mV, pulse amplitude: 25 mV, scan rate: 25 mV/s.

Samples	Voltammetric UA determination [a]			
	Spiked UA (µM)	Found UA (µM)	Recovery(%)	Relative standard deviation (%)
Artificial human urine	0	-	-	-
	10	10.30 ± 0.34	103.03	3.34
	25	26.08 ± 0.73	104.32	2.80
	50	51.89 ± 0.79	103.77	1.53
	80	78.96 ± 1.60	98.70	2.03
	100	100.19 ± 3.66	100.19	3.65
	130	126.60 ± 5.52	97.38	4.36
Human serum	0	288.29 ± 7.06	-	2.45
	10	298.75 ± 4.38	104.62	1.47
	25	313.24 ± 4.52	99.81	1.44
	50	338.25 ± 7.36	99.93	2.18
	80	368.86 ± 10.92	100.72	2.96
	100	393.05 ± 8.45	104.76	2.15
	130	414.08 ± 12.30	96.76	2.97

[a] All results are obtained by multiplying the relevant dilution factors stated in section 2.5.
